# Predicting Surgical Site Infection after Lumbar Laminectomy and Discectomy: A Cutting-edge Algorithmic Approach by Incorporating Ensembled Stacking into the Current State-of-the-art for Automated Machine Learning

**DOI:** 10.1007/s10143-025-03766-w

**Published:** 2025-09-18

**Authors:** Ali Haider Bangash, Kyle Mani, Samuel N. Goldman, Rose Fluss, Sertac Kirnaz, Ananth S. Eleswarapu, Mitchell S. Fourman, Yaroslav Gelfand, Saikiran G. Murthy, Reza Yassari, Rafael De la Garza Ramos

**Affiliations:** 1 Department of Neurosurgery, Montefiore Medical Center, Albert Einstein College of Medicine, 3316 Rochambeau Ave, Bronx, NY 10467 USA; 2 Albert Einstein College of Medicine, Bronx, NY USA; 3 Department of Orthopedic Surgery, Montefiore Medical Center, Albert Einstein College of Medicine, Bronx, NY USA

**Keywords:** Surgical site infection, Laminectomy, Discectomy, Automated machine learning, Ensemble, Stacking

## Abstract

To develop an algorithmic approach for predicting surgical site infections (SSIs) in patients undergoing lumbar laminectomy and discectomy for adult degenerative spinal disease (DSD) by incorporating ensembled stacking into state-of-the-art (SOTA) automated machine learning (aML). The study utilized a comprehensive dataset from a prospective multicenter surveillance study on SSIs following lumbar laminectomy and discectomy to manage adult DSD. The Google Colab environment was adopted to load the dataset using Python programming language. Nine algorithms, including eXtreme Gradient Boosting (XGBoost), Light Gradient Boosting Machine (LGBM), Neural Network (NN), Categorical Boosting (CatBoost), and Random Forest (RF), were adopted with hyperparameter tuning using the current SOTA for aML. Ensembling of the developed algorithmic models was carried out, followed by stacking and ensembled stacking. Five-fold stratified, shuffled cross-validation was implemented. The macro-weighted average Area Under the Receiver Operating Curve (mWA-AUROC) analysis was used to evaluate the discriminating classification ability of the developed models along with other evaluation metrics. A stacked ensemble algorithmic model, comprising a stacked XGBoost model and an ensemble of XGBoost, NN, CatBoost, LGBM, and RF algorithmic models, achieved an mWA-AUROC of 0.994, an accuracy of 98.7%, a sensitivity of 90% (95% CI: 68.30% – 98.77%) and a specificity of 98.81% (95% CI: 98.15% – 99.28%) upon predicting SSI. The top-weighted constituent model, XGBoost-20, identified operative time, smoking status, and patient age as the most significant predictors of SSI. We have made the development architecture of the algorithmic model available at GitHub for external validation. This study presented a novel algorithmic approach that integrated ensembled stacking into the current SOTA for aML to predict SSIs following lumbar laminectomy and discectomy procedures for adult DSD management. The performance of the stacked ensemble model highlighted its potential to serve as a valuable tool for clinicians, enabling more informed decision-making, optimized resource utilization, and enhanced patient outcomes in spine surgery. Future research should focus on validating the performance of the model in diverse clinical settings and exploring its integration into clinical practice.

**Supplementary Information:**

The online version contains supplementary material available at 10.1007/s10143-025-03766-w.

## Introduction

Surgical site infections (SSIs) following spinal procedures have a pooled incidence rate between 1.5 and 2.7% and are a significant source of morbidity due to the risk of reinfection [1, 2]. These postoperative complications can lead to prolonged hospital stays, increased treatment costs, and even patient mortality [3, 4]. Accurately predicting the risk of SSIs is crucial for implementing targeted preventive measures, optimizing patient management, and improving overall surgical outcomes [5].

Traditionally, clinicians have relied on traditional statistical models, such as logistic regression (LR), to identify risk factors and predict the likelihood of SSIs [6]. However, these conventional approaches may be limited in their ability to capture the complex, nonlinear relationships inherent in large, heterogeneous datasets [7]. The emergence of automated machine learning (aML) techniques offers a promising avenue to develop more robust and accurate predictive models for neurosurgical interventions [8].

In this study, we aimed to leverage the statistical capabilities of aML by incorporating ensembled stacking techniques into the current state-of-the-art (SOTA) for aML. The goal was to create a cutting-edge algorithmic model that could reliably predict the risk of SSIs following lumbar laminectomy and discectomy procedures for adult DSD management. By combining the strengths of multiple individual ML algorithmic models through ensembling and stacking, we hypothesized that the predictive performance of the output model would be superior to that of standalone algorithmic models.

## Methods

The study was carried out in accordance with the guidelines delineated under the principles of Helsinki including respecting the articles pertinent to patient consent. With the data shared after being completely de-identified, approval from an institutional review board was not sought. The TRIPOD Checklist for Prediction Model Development and Validation was adopted to report the findings of our study [9].

### Dataset

The dataset was shared by the prospective multicenter surveillance study on SSI following lumbar laminectomy and discectomy for adult DSD management collected between July, 2010 and June, 2024 with a minimum follow up of 1 month (Clinical trial number: not applicable) [10]. The data was collected from 10 tertiary care hospitals located in Japan’s Kantō region, and are part of the Greater Tokyo Area which is the world’s most populated metropolitan area [10]. The patients were included if they had more than 18 years of age and underwent posterior lumbar surgeries (laminectomy and/or discectomy) without fusion to manage DSD. Fusion surgery (irrespective of instrumentation), and surgical intervention for aetiologies other than DSD such as trauma or infection were excluded. The consecutive sampling technique was adopted with sample size power analysis calculations not reported to ascertain how the sample size was reached at [10].

A comprehensive set of patient and operative variables were collected including demographic factors such as age, sex, height, weight, and smoking status, as well as medical history indicators including diabetes mellitus and body mass index (BMI). Additionally, the dataset incorporated surgical details such as the American Society of Anesthesiologists score, surgical history in the operated area, steroid use, anatomic location of the surgery, type of operative procedure (with or without discectomy), emergency surgery status, presence of dural tear, utilization of endoscopic tubular surgery or operative microscope, operating time, and intraoperative bleeding. The dataset had no missing values.

SSI was diagnosed based on the Centers for Disease Control and Prevention definition, which categorized infections into superficial and deep SSI. Specific criteria encompassing purulent drainage, incision dehiscence with associated symptoms, presence of abscess, and clinical diagnosis by a healthcare provider (HCP) were used to confirm SSI within 30 days postoperatively [10]. Microbiological cultures were collected and recorded in the study by obtaining samples from all patients who developed SSI. In cases where patients underwent open debridement, microbiological cultures were taken to confirm the presence of SSI and guide further treatment decisions [10].

### Statistical analysis

Categorical variables were expressed as percentages whereas quantitative variables were expressed as mean (with range). Chi-square analysis was undertaken to appreciate the association of categorical variables with the development of SSI. Univariate LR analysis was undertaken to elucidate the association of continuous variables with SSI. A double-tailed *p*-value < 0.05 was recognized to be statistically significant. The conventional analysis was undertaken using Med Calc (version 20.215) and IBM SPSS (version 26).

By using Python programming language in the Google Colaboratory environment, the current SOTA for aML [11] was adopted to develop algorithmic models that could predict SSI in the said patient population. The dataset was loaded as a.CSV file with the categeorical variables as well as the outcome handled as binary data points (ZERO and ONE), whereas the continuous variables were handled as integers. The ‘compete’ mode was chosen with the ‘explain_level’ kept at 2 (the maximum value) that allowed for the development of learning curves, importance plots, and SHAP value plots, along with optimized tuning of hyper-parameters.

The first step in the development pipeline was to obtain preliminary insights into the dataset by implementing the simplest of algorithms. For the ‘compete’ mode, the Decision Trees algorithm was implemented which provided a simple decision rules tree with a maximum depth of 4 levels. The tree could be visualized via the ‘dtreeviz’ package. The second step was to concurrently develop models by individually adopting 7 algorithms including Extra Trees, Nearest Neighbors, eXtreme Gradiant Boosting (XGBoost), Light Gradiant Boosting Machine (LGBM), Neural Network (NN), Categorical Boosting (CatBoost) and Random Forest (RF)— each algorithmic model trained with default hyperparameters. For this step, one model each was developed by adopting each one of the 7 algorithms with each algorithm having a single set of default hyperparameter values for the prediction task (binary classification), independent of the loaded dataset.

The third step was to undertake a random search over defined set of individual hyperparameters for each one of the above mentioned 7 algorithms. The hyperparameters optimized for each one of the algorithms can be explored at https://github.com/mljar/mljar-supervised/tree/master/supervised/algorithms. For the fourth step, the individual hyperparameters of the best performing models for XGBoost, LGBM, and CatBoost algorithms obtained at the second and third steps were adopted to develop an algorithmic model each for these 3 algorithms where ‘golden features’ were developed by employing arithmetic functions on variables from the original dataset in a bid to enhance the predictive capability of the resulting algorithmic models.

The fifth step marked the initiation of feature selection is further broken down into two sub-steps. During the ‘random_feature’ sub-step, a new variable with a uniform distribution of positive and negative outcomes was introduced into the dataset. The extended dataset was adopted to train the algorithmic model outperforming all other models so far with its specific hyperparameters noted. A permutation-based feature importance graph was plotted to compare the performance of each variable of the original dataset against the randomly inserted novel variable in the extended dataset. The original variable was dropped if it did not perform well comparatively in at least half of the total 10 learners— A learner simply being the respective algorithmic model that iteratively improves by prioritizing misclassified cases, assigning them higher importance to rectify errors made by its predecessor models of the same algorithm [12]. During the ‘features_selection’ sub-step, the respective best performing algorithmic model each for 7 algorithms (Extra Trees, Nearest Neighbors, XGBoost, LGBM, NN, CatBoost, and RF) with the respective hyperparameters was trained using only selected features.

The fine-tuning of the algorithmic models was undertaken as the sixth step where two ‘hill_climbing’ sub-steps were undertaken to further refine models. During each ‘hill_climbing’ step, one randomly selected hyperparameter for each algorithmic model was fine-tuned with a variation introduced in its respective settings in both directions. It is impressed upon that the steps 2–6, which have been dissected empirically, were in fact undertaken in parallel and not longitudinally.

For the seventh step, all the developed algorithmic models were ensembled by calculating the respective weight values. For the eighth step, the prediction values obtained upon training algorithmic models on the original dataset were added to the said dataset, leading to the generation of the further extended dataset. The predictions of algorithmic models obtained upon training on the novel, extended dataset, along with the original dataset, were then fed into a meta-learner— stacked model— in a bid to improve accuracy. For the last step, an ensemble stacking was undertaken to combine the ensemble developed from algorithmic models trained on the original dataset, and the stacked model which was trained on the extended dataset including original dataset and stacked predictions [13].

Five-fold stratified, shuffled cross-validation was implemented for internal validation. Macro-weighted average Area Under the Receiver Operating Curve (mWA-AUROC) analysis was carried out to interpret the discriminating classification ability of the developed models in accordance with the schema outlined by Lau et al. [14]. Accuracy, log loss, precision, recall, and F1-score were also considered along with sensitivity, specificity, and positive as well as negative predictive values. The development architecture of the algorithmic model has been made available online as a Google Colab notebook on GitHub for future external validation.

## Results

The dataset consisted of 4,027 consecutive adult patients who underwent lumbar laminectomy and discectomy for adult DSD management in ten Japanese hospitals from July 2010 to June 2014. The mean age of the patients being 59.2 years (18–94 years) with 31% (*n* = 1235) being female (Table 1).

**Table 1:** Demographic characteristics and surgical factors in patients undergoing lumbar laminectomy and discectomy for adult degenerative spinal disease management

Demographics & variables	Data
Total patients	4027
Gender	Female: 1235 (30.6%), Male: 2792 (69.4%)
Mean age (with range)	59.2 years (18–94 years)
Smoking status	Present in 1006 cases (25%)
Diabetes mellitus	Present in 606 cases (15%)
Body mass index	Mean: 24.8 kg/m^2^
ASA score	Mean: 1.8
Surgical history	Present in 604 cases (15%)
Steroid use	Present in 322 cases (8%)
Anatomic location	Lumbar spine only in 3020 cases (75%) Including sacrum (L5/S1) in 1007 cases (25%)
Operative procedure	Discectomy in 2416 cases (60%)
Emergency surgery	Present in 201 cases (5%)
Dural tear	Present in 403 cases (10%)
Endoscopic tubular surgery	Used in 1208 cases (30%)
Operative microscope	Used in 805 cases (20%)
Operating time	Mean: 2.5 h
Intraoperative bleeding	Mean: 150 ml

*ASA* American Society of Anesthesiologists; *SSI* Surgical site infection

1% (*n* = 26) of patients suffered from an SSI. An overwhelming 88.5% of those who suffered from an SSI (*n* = 23/26) were males (χ2 = 4.504; *p*-value = 0.034). SSI rates were not significantly affected by comorbid diabetes (χ2 = 1.616; *p*-value = 0.204) or smoking behaviors (χ2 = 2.889; *p*-value = 0.089). Steroids (χ2 = 1.912; *p*-value = 0.167), hemodialysis (χ2 = 0.322; *p*-value = 0.57) or preoperative ASA score (χ2 = 2.44; *p*-value = 0.486) didn’t influence SSI rates significantly, either.

SSI rates were not found to be significantly affected by undertaking discectomy (χ2 = 3.675; *p*-value = 0.05) or an iatrogenic dural tear (χ2 = 1.12; *p*-value = 0.277). The inclusion of L5/S1 level (χ2 = 0.014; *p*-value = 0.905) and revision surgery (χ2 = 2.695; *p*-value = 0.101) did not affect SSI rates significantly, either.

84.6% of those who developed an SSI (*n* = 22/26) were not surgically intervened endoscopically (χ2 = 6.372; *p*-value = 0.01). However, the use of a microscope was not found to affect SSI rates significantly (χ2 = 0.482; *p*-value = 0.487). Moreover, bio-clean room (χ2 = 0.359; *p*-value = 0.549), emergent nature of surgery (χ2 = 1.29; *p*-value = 0.256), or administration of Cefazolin (χ2 = 0.517; *p*-value = 0.472) were also found not to significantly influence SSI rates.

LR analysis yielded only the use of the endoscope tubular approach to be significantly associated with SSI [OR = 0.27 (95% CI: 0.08–0.94); Coefficient= −1.28128; Standard error = 0.62267; *p*-value = 0.0396].

**Fig. 1:**
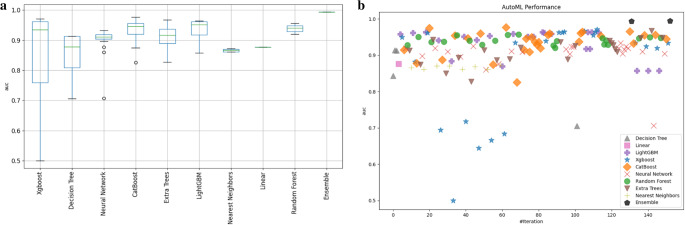
Stacked ensemble algorithmic model outperforming individual algorithmic models in prediction of surgical site infection after lumbar laminectomy and discectomy for adult degenerative spinal disease management. **a** The boxplot which displayed the distribution of the area under the curve (AUC) values for different machine learning models, including XGBoost, Decision Tree, Neural Network, CatBoost, Extra Trees, LightGBM, Nearest Neighbors, Linear, Random Forest, and the Ensemble algorithmic model. The Ensemble algorithmic model demonstrated the highest and most consistent performance; and the (**b**) Scatter Plot that illustrated the AUC performance across multiple iterations for various models in the AutoML framework. Each symbol represents a different model, as indicated in the legend. The Ensemble model consistently achieved superior AUC scores compared to individual algorithmic models

**Fig. 2:**
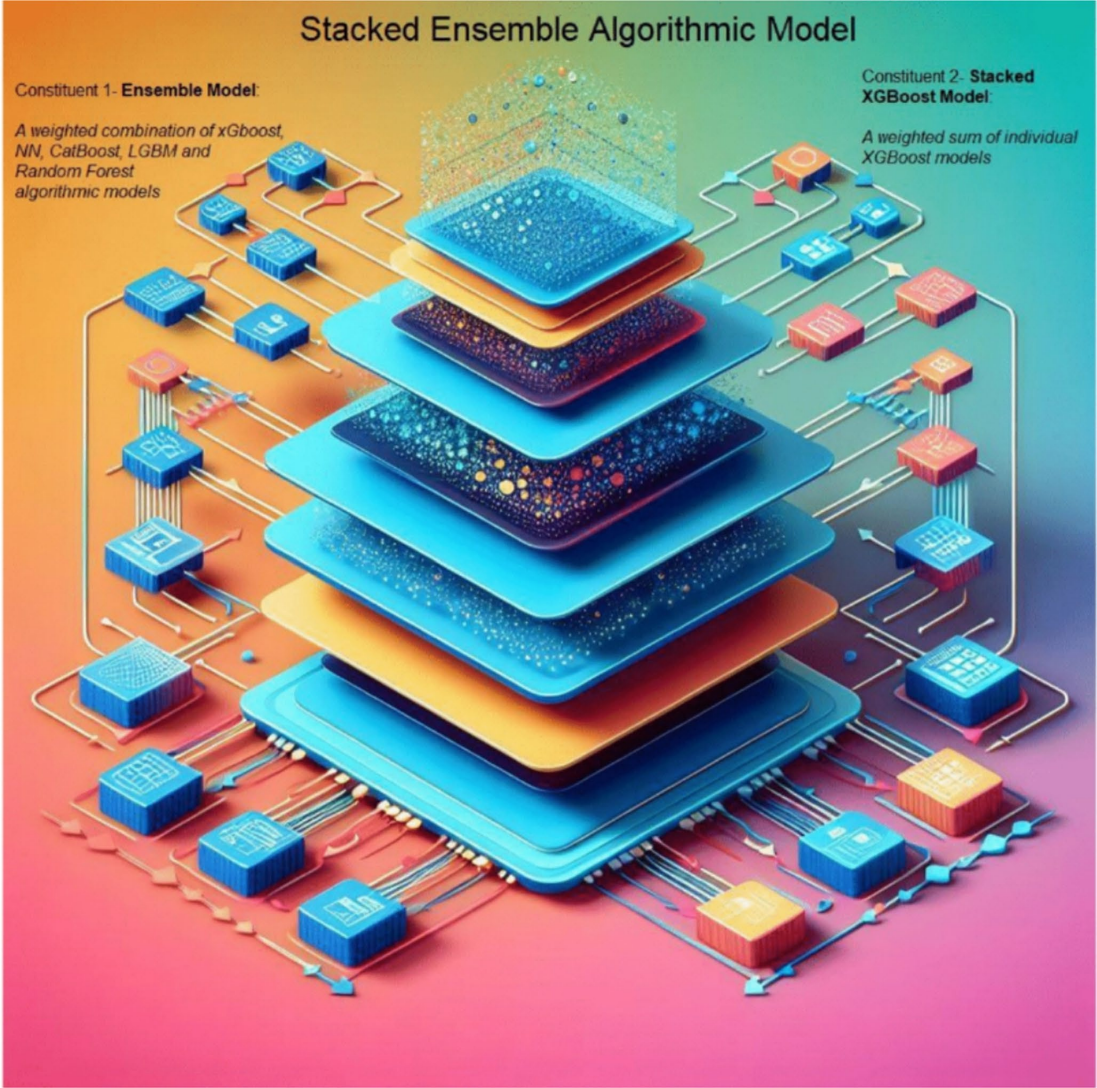
Architecture of the stacked ensemble algorithmic model for predicting surgical site infection after lumbar laminectomy and discectomy. This figure illustrates the two key constituents of the stacked ensemble algorithmic model: Ensemble Model (left): A weighted combination of multiple machine learning models, including XGBoost, Neural Networks (NN), CatBoost, LightGBM (LGBM), and Random Forest; Stacked XGBoost Model (right): A weighted sum of individual XGBoost models. The diagram visually represents how different machine learning models are integrated within the ensemble framework to enhance predictive performance

**Fig. 3:**
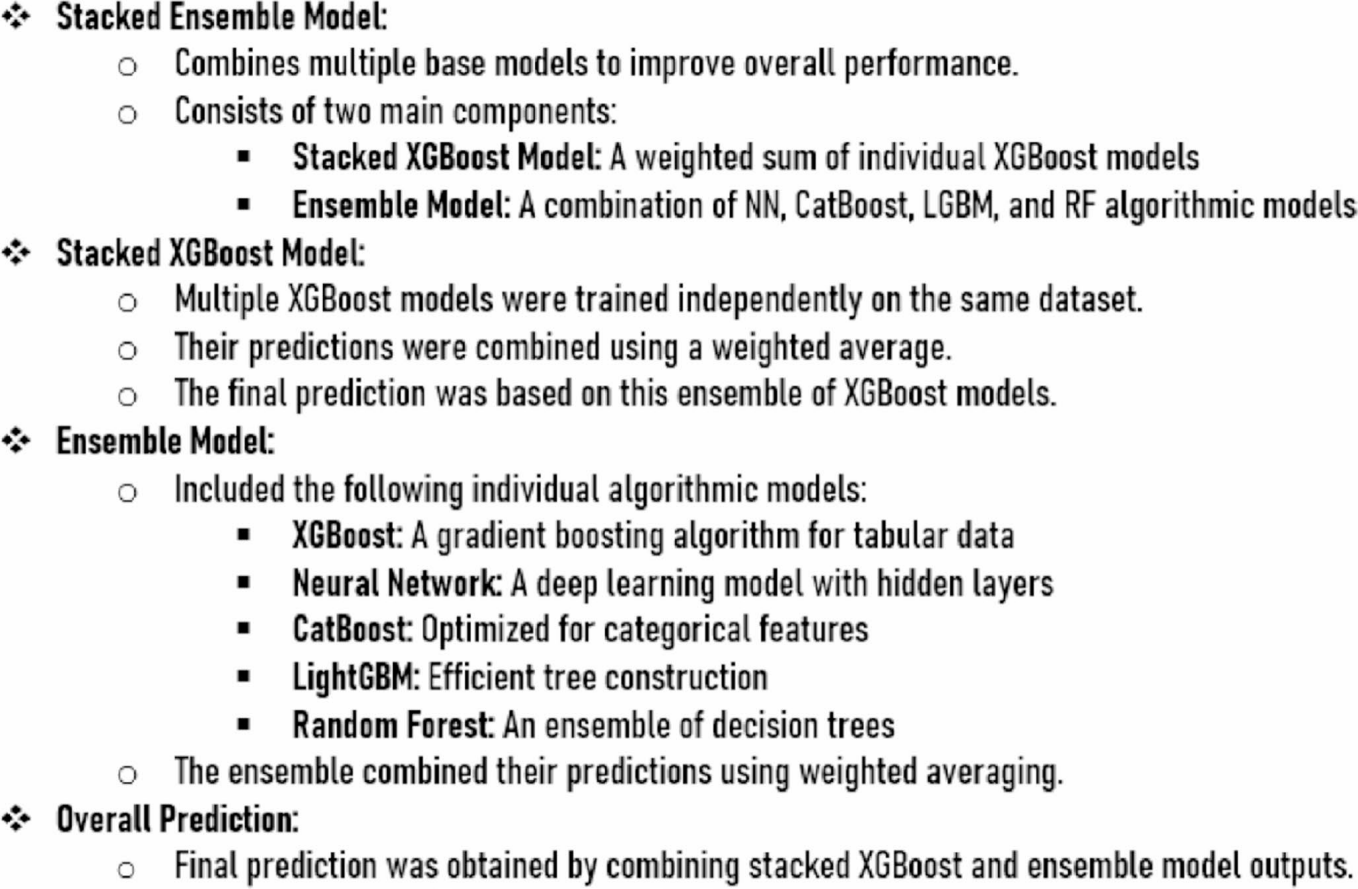
Predictive workflow of the stacked ensemble algorithmic model for surgical site infection prediction after lumbar laminectomy and discectomy for adult degenerative spinal disease management. This flowchart outlines the step-by-step process adopted by the stacked ensemble model to generate predictions. Stacked Ensemble Model: Combines multiple base models to enhance overall predictive performance. It comprises of: Stacked XGBoost Model: Multiple XGBoost models are independently trained on the same dataset, and their predictions are aggregated using a weighted average. Ensemble Model: Integrates predictions from multiple machine learning algorithms, including XGBoost, Neural Networks, CatBoost, LightGBM, and Random Forest, using a weighted averaging approach. Final Prediction: The outputs of both the stacked XGBoost and ensemble models are combined to produce the final prediction for surgical site infection after lumbar laminectomy and discectomy

Upon implementing the current SOTA for aML in Google Colab on the entire dataset, a stacked ensemble algorithmic model came up as the one with the most robust performance metrics amongst all of the trained algorithmic models (Fig. 1). The stacked ensemble algorithmic model constituted of a stacked XGBoost algorithmic model and an ensemble of XGBoost, NN, CatBoost, LGBM, and RF algorithmic models (Fig. 2). The prediction flowchart illustrates how a prediction was made by the developed stacked ensemble algorithmic model (Fig. 3). The stacked ensemble algorithmic model predicted SSI amongst adult patients managed with lumbar laminectomy and discectomy for DSD with an mWA-AUROC of 0.994 and an accuracy of 98.7% (Table 2).

**Table 2:** Performance metrics of the stacked ensemble algorithmic model predicting surgical site infection after lumbar laminectomy and discectomy for adult degenerative spinal disease management

Performance metrics	
AUROC	0.99
Accuracy	98.7%
Log loss	0.12
F1 score	0.63
Precision	48.64%
Recall	100%
MCC	0.65
Sensitivity	90% (95% CI: 68.30% − 98.77%)
Specificity	98.81% (95% CI: 98.15% − 99.28%)
Positive likelihood ratio	75.88 (95% CI: 47.42–121.44)
Negative likelihood ratio	0.10 (95% CI: 0.03–0.38)
Positive predictive value	48.65% (95% CI: 37.18% − 60.26%)
Negative predictive value	99.87% (95% CI: 99.53% − 99.97%)

**Fig. 4:**
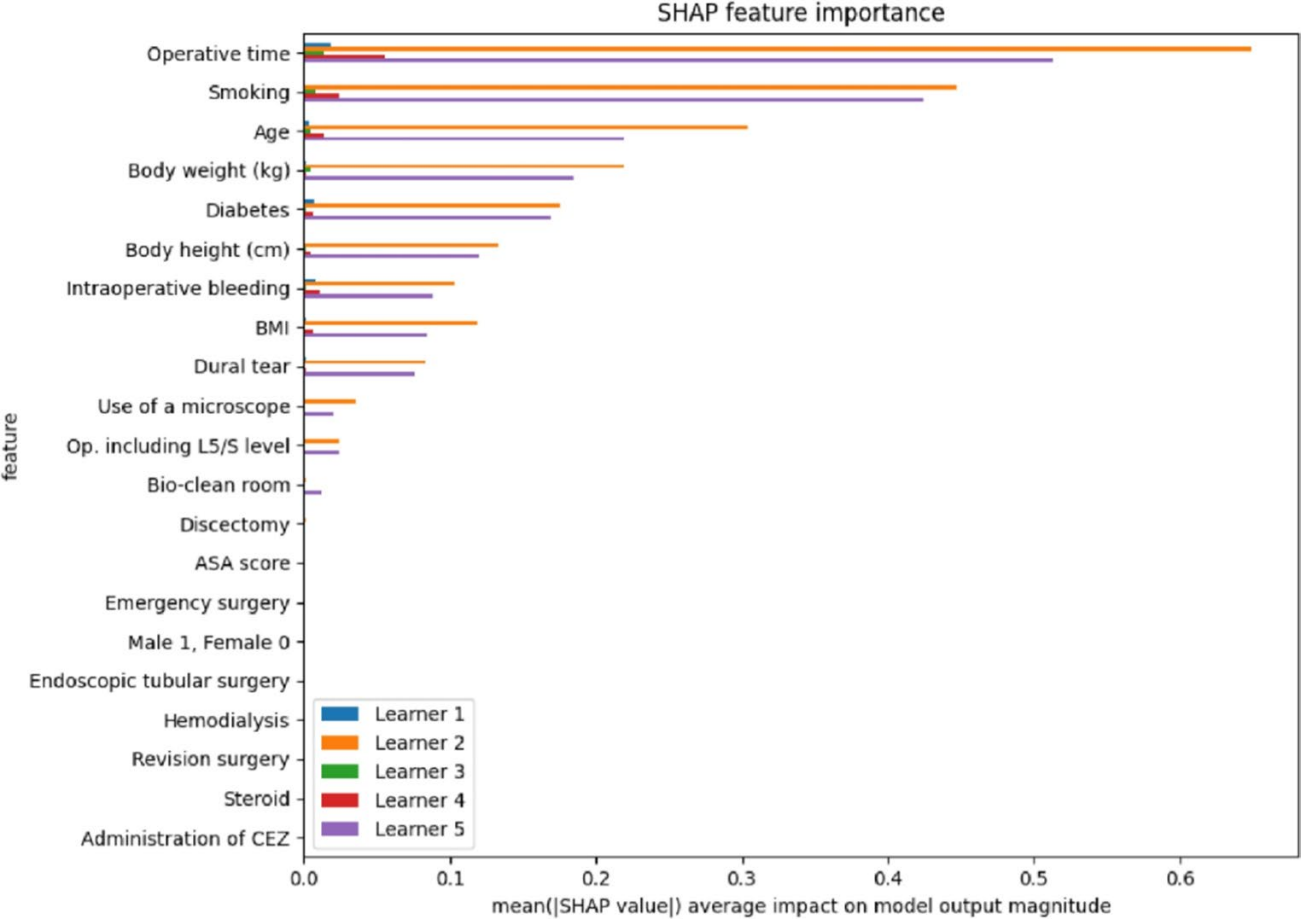
SHAP-based feature importance in the XGBoost-20 model for predicting surgical site infection after lumbar laminectomy and discectomy for adult degenerative spinal disease management. This bar plot illustrates the SHAP (SHapley Additive exPlanations) feature importance values, highlighting the contribution of different clinical and surgical variables to the prediction of surgical site infection after lumbar laminectomy and discectomy. Features are ranked based on their mean SHAP values, which represent their average impact on model output magnitude. Top Predictive Features: Operative time, smoking status, and age have the highest influence on the model’s predictions. Multiple Learners: The contributions of individual learners within the XGBoost-20 model are represented using different colors. This analysis helps interpret the decision-making process of the model by identifying key risk factors associated with surgical site infection after lumbar laminectomy and discectomy for adult degenerative spinal disease management

Going through the performance evaluation metrics of constituent models suggested that the stacked XGBoost algorithmic model had the highest accuracy and MCC score whereas the ensemble algorithmic model had the highest AUROC (Table 3). Amongst the top 3 most-weighted constituent algorithmic models composing the Ensemble algorithmic model, the XGBoost-20 algorithmic model^1^ outperformed its competitors by leading in 10 out of 13 performance evaluation metrics (Table 4). The XGBoost-20 algorithmic model recognized operative time, along with smoking status and age of the patients, as the most significant predictor of SSI (Fig. 4).

**Table 3:** Individual performance metrics of selected constituent algorithmic models of the stacked ensemble model predicting surgical site infection after lumbar laminectomy and discectomy for adult degenerative spinal disease management

Performance metrics	Stacked XGBoost	Ensemble
AUROC	0.92	**0.99**
Accuracy	**99.26%**	98.7%
Log loss	0.14	**0.11**
F1 score	**0.75**	0.63
Precision	**64%**	48%
Recall	**100%**	**100%**
MCC	**0.75**	0.65
Sensitivity	**90% (95% CI: 68.3% − 98.77%)**	**90% (95% CI: 68.3% − 98.77%)**
Specificity	**99.38% (95% CI: 98.86% − 99.7%)**	98.81% (95% CI: 98.15% − 99.28%)
Positive likelihood ratio	**144.18 (95% CI: 76.41–272.04)**	75.88 (95% CI: 47.42–121.44)
Negative likelihood ratio	**0.1 (95% CI: 0.03–0.37)**	**0.1 (95% CI: 0.03–0.38)**
Positive predictive value	**64.29% (95% CI: 48.82% − 77.25%)**	48.65% (95% CI: 37.18% − 60.26%)
Negative predictive value	**99.87% (95% CI: 99.53% − 99.97%)**	**99.87% (95% CI: 99.53% − 99.97%)**

**Table 4:** Individual performance metrics of top 3 most-weighted constituent algorithmic models composing the ensemble algorithmic model predicting surgical site infection after lumbar laminectomy and discectomy for adult degenerative spinal disease management

Performance metrics	XGBoost − 15^1^	XGBoost − 20^1^	Random forest − 45^1^
AUROC	0.5	0.93	**0.94**
Accuracy	79.28%	**98.46%**	98.02%
Log loss	0.14	0.08	**0.04**
F1 score	0.02	**0.56**	0.42
Precision	1.23%	**43%**	33%
Recall	**100%**	**100%**	95%
MCC	< 0.001	**0.58**	0.45
Sensitivity	20% (95% CI: 5.73% − 43.66%)	**80% (95% CI: 56.34% − 94.27%)**	60% (95% CI: 36.05% − 80.88%)
Specificity	80.02% (95% CI: 77.98% − 81.96%)	**98.69% (95% CI: 98% − 99.19%)**	98.5% (95% CI: 97.78% − 99.04%)
Positive likelihood ratio	1 (95% CI: 0.41–2.42)	**61.03 (95% CI: 37.84–98.44)**	40.05 (95% CI: 23.47–68.35)
Negative likelihood ratio	1 (95% CI: 0.8–1.25)	0.2 (95% CI: 0.08–0.49)	**0.41 (95% CI: 0.24–0.69)**
Positive predictive value	1.23% (95% CI: 0.51% − 2.93%)	**43.24% (95% CI: 32.08% − 55.14%)**	33.3% (95% CI: 22.66% − 46.04%)
Negative predictive value	98.77% (95% CI: 98.47% − 99.01%)	**99.75% (95% CI: 99.4% − 99.89%)**	99.5% (95% CI: 99.14% − 99.7%)

^1^The numerical “15”, “20”, and “45” are adopted by the MLjar library arbitrarily to differentiate between different model architectures adopting the same algorithm but varying hyperparameter tuning variables

The development architecture of the algorithmic model has been made available as a Google Colab notebook on GitHub (https://github.com/hhaider15/SSI-stacked-ensemble-predictive-algorithmic-model.git*)* for future external validation.

## Discussion

SSIs are a significant complication following lumbar laminectomy and discectomy that is routinely performed for managing adult DSD. The reported prevalence rates for SSIs in lumbar surgeries varied widely, ranging from 0.7 to 16%, depending on factors such as patient comorbidities, surgical technique, and perioperative management [15]. SSIs can lead to serious complications, including prolonged hospital stays, increased healthcare costs, readmissions, and, in severe cases, hardware failure or the need for revision surgeries [16]. These infections have also been reported to impair functional outcomes by delaying recovery and increasing pain and disability [17]. Addressing SSIs in this patient population is crucial as the degenerative nature of the spinal pathology often predisposes patients to the prolonged requirement of management [18], potentially compounding the impact of infection on their quality of life. Additionally, a substantial proportion of patients undergoing lumbar surgeries are older adults with comorbidities, further increasing the risk of infection [19]. Effective preventive strategies, such as strict aseptic techniques, perioperative antibiotic administration, and optimal postoperative care, are essential to minimize infection risks and improve clinical outcomes [20].

The findings of this study demonstrated the successful development of an accurate and reliable predictive model for SSIs following lumbar laminectomy and discectomy procedures to manage adult DSD. The stacked ensemble algorithmic model, which combined a stacked XGBoost model and an ensemble of XGBoost, neural network, CatBoost, LGBM, and RF models, achieved an mWA-AUROC of 0.994 and an accuracy of 98.7%.

The superior performance of the stacked ensemble model can be attributed to its ability to leverage the complementary strengths of multiple individual algorithms through the ensembling and stacking techniques. By integrating the predictive capabilities of these diverse models, the stacked ensemble was able to capture complex nonlinear relationships and effectively handle the inherent heterogeneity in the dataset. The high sensitivity (90%) and specificity (98.81%) of the stacked ensemble model demonstrated its ability to accurately identify both true positive and true negative cases, recognizing it as a valuable potential tool for clinical decision-making.

A strong negative predictive value (99.87%) of the ensemble stacked algorithmic model suggested that it can reliably rule out the risk of SSI, potentially reducing unrequired implementation of SSI antibiotic protocol, which would lead to potentially decreased exposure to the risks associated with antibiotics such as Gentamycin. It would also potentially reduce antibiotic resistance burden and optimize resource utilization. A more targeted approach to implementing the SSI antibiotic protocol shall potentially provide better outcomes by potentially diminuting the risks associated with antibiotic provision. Furthermore, the XGBoost-20 algorithmic model, which was one of the top-weighted constituents of the ensemble, identified operative time, smoking status, and patient age as the most influential predictors of SSI. This finding, which is consistent with previous studies that have highlighted the importance of these factors in the development of SSIs following spinal procedures [6], reinforced the significance of the physiological reserve of a patient’s body to withstand the stress of surgical intervention to be considerably more influential in protecting the patient from an SSI than other intraoperative variables such as the use of the operative endoscope. These findings also have several direct implications for clinical practice.

The prominence of operative time as a predictor suggests that surgical efficiency should be prioritized without compromising surgical quality. Clinically, this supports the implementation of standardized surgical protocols, optimal operating room preparation, and surgeon training programs focused on technical efficiency [21]. For complex cases where extended operative times are anticipated, enhanced preventive measures such as additional antibiotic redosing and meticulous hemostasis may be warranted. Institutions might consider implementing time-based risk stratification, with cases exceeding certain duration thresholds automatically qualifying for enhanced SSI prevention protocols.

Moreover, the identification of smoking as a key predictor reinforces the importance of preoperative smoking cessation programs [22]. Spine surgeons should, therefore, consider implementing mandatory smoking cessation at least 4 weeks prior to elective procedures when possible [23]. In case of urgent surgical intervention in a patient who is an active smoker, heightened vigilance and potentially more aggressive prophylactic measures may be justified. This finding also supports the development of risk-stratified approaches where active smokers receive enhanced wound care education and closer postoperative monitoring.

In addition to this, the significance of age as a predictor highlighted the need for age-appropriate perioperative care modifications. Older patients may benefit from preoperative nutritional optimization, careful medication management to avoid immunosuppression, and potentially extended antibiotic prophylaxis in selected cases [24]. The patient’s age should be incorporated into preoperative risk assessment tools and may warrant consideration when planning the extent and duration of surgery.

The relative importance of these patient-specific factors over procedure-related variables (such as the use of the endoscope) suggested that the optimization of the perioperative management protocols may yield greater benefits for SSI prevention than modifications to surgical technique alone [25]. This finding challenges the common focus on intraoperative factors and supports a more holistic approach to SSI prevention that begins well before the patient enters the operating room. These findings could be translated into a risk-stratification protocol where patients with multiple high-risk features (advanced age, smoking history, and anticipated lengthy procedures) receive enhanced preventive interventions. Such interventions should also include preoperative patient comorbidity optimization, chlorhexidine shower before surgery, hair clipping outside of the operating room, advanced wound closure techniques, extended antibiotic prophylaxis, modifications to post-surgical dressing practices, and more intensive postoperative monitoring. Additionally, these findings suggest that resource allocation for SSI prevention might be optimized by focusing on patient-specific risk factors rather than the universal application of costly interventions.

ML is being extensively explored for its predictive capabilities in the context of spine surgery, offering a transformative approach to enhancing patient safety and optimizing surgical outcomes. By harnessing advanced algorithms and predictive analytics, clinicians can proactively identify potential risks, predict outcomes, and personalize treatment strategies to improve surgical interventions and patient satisfaction [26]. Chen et al. adopted LR, LASSO regression, support vector machine, and RF algorithms to identify four predictors of SSI: Modic changes, sebum thickness, hemoglobin, and glucose. They, then, developed a conventional model that demonstrated excellent performance, with an AUROC curve of 0.988 in the test group and 0.987 in the validation group [27]. In contrast, our ensemble stacked algorithmic model presented a more advanced and cutting-edge algorithmic approach that incorporated ensembled stacking techniques, resulting in a stacked ensemble algorithmic model that outperformed the model developed by Chen et al. with a superior mWA-AUROC of 0.994 and an accuracy of 98.7%. Moreover, Lu et al. reported that the RF model achieved the highest AUROC (0.9916), specificity (99%), and precision (97%), while the Gradient Boosting model achieved the highest sensitivity (95%) and the K-Nearest Neighbors model achieved the highest sensitivity (99%) for predicting SSI after posterior cervical surgery [28]. In contrast, the stacked ensemble approach adopted by us, which combined multiple advanced algorithms like XGBoost, Neural Network, CatBoost, LGBM, and RF, enabled the model to capture more complex relationships in the data compared to the individual models evaluated by Lu et al. as ensembling and stacking techniques are generally known to improve the robustness and predictive performance of ML models compared to standalone algorithms [29].

The innovative use of ensemble and stacking methods to leverage the strengths of multiple ML algorithms has led to the development of a superior predictive model that can more accurately identify patients at risk of SSIs following lumbar laminectomy and discectomy procedures to manage adult DSD, surpassing the strong performance of the previously developed predictive algorithmic models. Zhang et al. forecasted SSI subsequent to spine surgery through the application of ML algorithms. The Naïve Bayes (NB) algorithmic model stood out as the most efficient among the models assessed, showcasing an average AUC of 0.96, sensitivity of 78%, specificity of 88%, and accuracy of 87%. Key variables within the NB model encompassed age, BMI, smoking, cerebrospinal fluid leakage, drain duration, and pre-operative albumin level [30]. Multiple ML algorithmic models were developed and validated by Wang et al. to predict the risk of SSI following minimally invasive transforaminal lumbar interbody fusion under the Quadrant channel [31]. The researchers found that the NB model demonstrated the highest performance in predicting SSI, with an average AUC of 0.78, sensitivity of 93%, specificity of 82%, and accuracy of 90% in the whole dataset [31]. The relative importance of variables in the NB model was determined, with pre-operative HbA1c, estimated blood loss, operation time, pre-operative albumin, BMI, and age being the high-ranking variables in descending order of importance [31]. Our stacked ensemble model outperformed both aforementioned NB models across all comparable performance metrics other than the sensitivity of the NB model developed by Wang et al. [31].

The implementation of the current SOTA for aML, coupled with the incorporation of ensembled stacking techniques, represented a cutting-edge approach in the field of SSI prediction for spine surgery. By leveraging the power of these advanced ML methods, the developed model can potentially enhance clinical decision-making, guide targeted preventive strategies, and ultimately improve patient outcomes—both generally [32] and in the specific context of spine surgery [33]. While our model identifies known risk factors (operative time, smoking status, and age), its practical value lies in the precision with which it quantifies individual patient risk, enabling truly personalized preventive strategies. In clinical practice, this algorithm could be implemented as a point-of-care decision support tool integrated into electronic health records. Preoperatively, surgeons could use the model to identify high-risk patients who would benefit from targeted interventions such as more aggressive smoking cessation programs, nutritional optimization, or consideration of minimally invasive approaches to reduce operative time. Intraoperatively, the model could inform decisions about antibiotic redosing protocols, wound closure techniques, and drain placement. Postoperatively, resources for wound monitoring and follow-up could be allocated based on individualized risk profiles, with high-risk patients receiving more intensive surveillance. This personalized approach represents a shift from current one-size-fits-all prevention strategies to precision medicine in surgical infection prevention. While this study focused specifically on SSI prediction following lumbar procedures, the methodological framework that we have developed has broader implications for diagnostic and predictive modeling across various clinical domains. The ensemble stacking approach demonstrated here could be adapted to predict other postoperative complications including but not limited to thromboembolic events, surgical complications, and medical complications. Beyond complications, this framework could also potentially be applied to predict clinical outcomes, length of stay, readmission risk, and response to specific interventions. Specifically, similar approaches could be developed to predict cerebrospinal fluid leaks, neurological deficits, and hardware failure. The ability to leverage multiple algorithms through ensembling and stacking techniques allows for capturing complex relationships in clinical data that might be missed by traditional statistical methods or single-algorithm approaches [34], making this methodology particularly valuable for conditions with multifactorial etiologies and heterogeneous patient populations.

While the stacked ensemble model demonstrated exceptional performance, it is important to acknowledge the limitations of this study. The dataset adopted for the analysis was imbalanced as only 1% (*n* = 26) of patients developed an SSI. Imbalanced datasets introduce several risks, including bias towards the majority class, which can lead to poor predictive performance for the minority class that often represents critical outcomes [35]. They can also result in misleading performance metrics, such as accuracy, which may give a false sense of model effectiveness while masking inadequate learning from the minority class [35]. Furthermore, the developed stacked ensembled algorithmic model may struggle to generalize to new data with different class distributions, leading to unreliable predictions. This imbalance can also increase the rates of false positives and false negatives, which can have serious implications in clinical decision-making [35].

The dataset was also limited to a specific geographic region and time period, which may not fully capture the diversity of patient populations and surgical practices across different healthcare settings. Validating the model’s performance on external datasets from diverse clinical settings would further strengthen the generalizability of the findings [36]. Additionally, the study focused on predicting SSIs following lumbar laminectomy and discectomy procedures for adult DSD management. Future research could explore the applicability of this approach to other spinal procedures and aetiologies, such as fusion surgeries and oncological ailments, to broaden the scope and utility of the predictive model. Another potential area for future investigation is the integration of the developed predictive model into clinical workflows and decision support systems. Evaluating the impact of the developed model on clinical decision-making, patient outcomes, and resource utilization in a real-world setting shall provide valuable insights into its practical implications and potential for widespread adoption [37]. Although the current study did rely on a prospectively calculated dataset, more comprehensive data, including intraoperative details and postoperative surveillance data, such as iatrogenic durotomy and postoperative cerebrospinal fluid leak, that have not been reported in the adopted dataset but are known to influence SSI rates [38], could enhance the predictive capabilities of the predictive algorithmic model and provide better risk prediction [39]. Therefore, we plan to externally validate our developed algorithmic model in the near future on the comprehensive prospectively collected SSI tracking database maintained in our institution. We also plan to further optimize our institution’s SSI protocol by exploring if the current ‘high-risk’ criteria for SSI require a stringent revision after externally validating the developed model on the prospectively calculated database.

To promote transparency and encourage further validation and development of our models, we have made our development architecture of the algorithmic model available on GitHub (https://github.com/hhaider15/SSI-stacked-ensemble-predictive-algorithmic-model.git*).* This shall allow other researchers and clinicians to easily implement and test our model, potentially accelerating the translation of these findings into clinical practice and facilitating the external validation process using larger, multi-center cohorts.

## Conclusion

This study presented a novel algorithmic approach that integrated ensembled stacking techniques into the current SOTA for aML to predict SSIs following lumbar laminectomy and discectomy procedures for adult DSD management. The exceptional performance of the stacked ensemble algorithmic model highlighted its potential to serve as a potentially valuable tool in the arsenal of clinicians and HCPs, empowering them to make well-informed decisions, optimize resource utilization, and enhance the overall quality of care for patients being surgically managed for DSDs. Future research should focus on validating the performance of the developed algorithmic model in diverse clinical settings, exploring its applicability to other spinal procedures, and evaluating its integration into clinical practice to further advance the field of SSI prediction and prevention in spine surgery.

## Supplementary Information

Below is the link to the electronic supplementary material.

Supplementary Material 1 (DOCX 218 KB)

## Data Availability

Data is provided within the manuscript and related files.

## Footnotes

1 The numerical “20” is adopted by the MLjar library arbitrarily to differentiate between different model architectures adopting the same algorithm but varying hyperparameter tuning variables.
